# Polymorphism of Myostatin Gene in Intron 1 and 2 and Exon 3, and Their Associations with Yearling Weight, Using *PCR-RFLP* and *PCR-SSCP* Techniques in Zel Sheep

**DOI:** 10.1155/2012/472307

**Published:** 2012-06-20

**Authors:** Elena Dehnavi, Mojtaba Ahani Azari, Saeed Hasani, Mohammad Reza Nassiry, Mokhtar Mohajer, Alireza Khan Ahmadi, Leila Shahmohamadi, Soheil Yousefi

**Affiliations:** ^1^Department of Animal Science, Gorgan University of Agricultural Sciences and Natural Resources, P.O. Box 4913815739, Gorgan, Iran; ^2^Department of Animal Science, Ferdowsi University of Mashhad, P.O. Box 9177948978, Mashhad, Iran; ^3^Golestan Agriculture Jahad, P.O. Box 49174, Gorgan, Iran; ^4^Department of Animal Science, Faculty of Agrecultural Scinece and Natural Recources, Gonbad University, P.O. Box 4971799151, Gonbad, Iran

## Abstract

The aim of present study was to investigate myostatin gene polymorphism and its association with yearling weight records in Zel sheep using *PCR-RFLP* and *PCR-SSCP* methods. Blood samples were collected from 200 Zel sheep, randomly, and DNA was extracted using modified salting out method. Polymerase chain reaction was carried out to amplify 337, 222, and 311 bp fragments, respectively, comprising a part of exon 3, intron 1, and intron 2 of myostatin gene. In addition, exon 3 was digested by *HaeIII* enzyme under *RFLP* method, and introns 1 and 2 were studied using *SSCP*. Under *RFLP* method, all samples showed *mm* genotype. Under *SSCP* method, intron 1 was also monomorph but intron 2 was polymorph (AA, AB, and BB). The allelic frequencies for *A* and *B* were 75.5 and 24.5%, respectively. This locus was not in Hardy-Weinberg equilibrium (*P* < 0.05), and there was no significant effect of myostatin gene on yearling weights.

## 1. Introduction

Considerable progress in farm animal breeding has been made in the last few decades, but achieving greater understanding in the improvement of meat quality was very slow before molecular markers became an accessible technology with wide applications in breeding methods [1].

Meat quality is one of the important economic traits in domestic animals. Determination of meat quality requires analysis and classification of fat content, composition, tenderness, water-holding capacity, color, oxidative stability, and uniformity. Meat quality is affected by several factors such as breed, genotype, feeding, fasting, preslaughter handling, stunning, slaughter methods chilling, and storage conditions [2].

Finding of main genes responsible for meat quality will benefit the producers. In recent years, a lot of works have been performed in this field to find potential genes or chromosome regions associated with the meat quality traits in different farm animals, including cattle, sheep, and chicken. Myostatin (*MSTN*) or growth differentiation factor-8 (GDF-8) is a member of the mammalian growth transforming family (TGF-beta superfamily), which plays an important role in the regulation of embryonic development and tissue homeostasis in adults [3]. They are known to block myogenesis, hematogenesis and enhance chondrogenesis as well as epithelial cell differentiation in vitro. In mice, null mutants are significantly larger than wild-type animals, with 200–300% more skeletal-muscle mass, because of hyperplasia and hypertrophy [4]. Muscular hypertrophy (mh), also known as “double-muscling” in cattle, has been recognized as a physiological character for years [5] and is seen in Belgian Blue and Piedmontese cattle [6]. These animals had less bone, less fat, and 20% more muscle on an average [7]. Mutations within myostatin gene were red to muscular hypertrophy allele (mh allele) in the double muscle breeds [6]. Such a major effect of a single gene on processing yields opened a potential channel for improving processing yields of animals using knockout technology [8]. Therefore, considering of myostatin gene in farm animals is important to find better animal which opens interesting prospects for future selection programs, especially marker-assistant selection for economic traits.

In Iran, sheep meat is a major source of animal protein and investigation for meat quality and related genes is important. Zel sheep is a native Iranian meat breed and plays a great role in sheep rearing activities in the north of Iran [9]. The aim of present study was to identify genotypes of myostatin gene and their association with yearling weight records in Zel sheep using *PCR*-*RFLP* and *PCR*-*SSCP* methods in order to find effective alleles influencing meat quantity and quality traits in sheep.

## 2. Materials and Methods

### 2.1. Animals and DNA Extraction

Blood samples were randomly collected from 200 (190 ewes and 10 rams) Zel sheep (there were 230 ewes and 30 rams in this station) from Shirang's Zel Breeding Station in Fazel Abad city of Golestan province. DNA was extracted from 3 mL of blood as described by Miller et al. [10]. Quality and quantity of DNA were measured by visual and spectrophotometer methods. 

### 2.2. *PCR*

Two pairs of primers were designed for each of intron 1 and 2 and exon 3 regions. The primer sequences are presented in Table 1. An aliquot of 100 ng genomic DNA was amplified in a total volume of 15 *μ*L *PCR* mix. The *PCR* mix consisted of 7.5 *μ*L Master mix (Cinna clon), 2 *μ*L forward and reverse primers (10 pmol/*μ*L), and 4.5 *μ*L ddH2O. Amplification conditions are shown in Table 2.

In every experiment, negative controls were used, aiming to avoid contaminations. Assays were performed in a thermal cycler (Personal Cycler-Biometra, CA, German), and the amplicons were analyzed by 1.5% agarose gel electrophoresis. The gels were stained with ethidium bromide and visualized under ultraviolet light.

### 2.3. Digestion Reaction

10 *μ*L of *PCR* products were incubated for 10 h at 37°C with 1 *μ*L (10 units) of *HaeIII* enzyme for myostatin gene (just for exon 3, using *RFLP* method). Digestion products were separated by electrophoresis on 8% nondenaturing polyacrylamide gels, stained by silver nitrate staining method [11] (Figure 3).

### 2.4. *SSCP*

**Figure 1:**
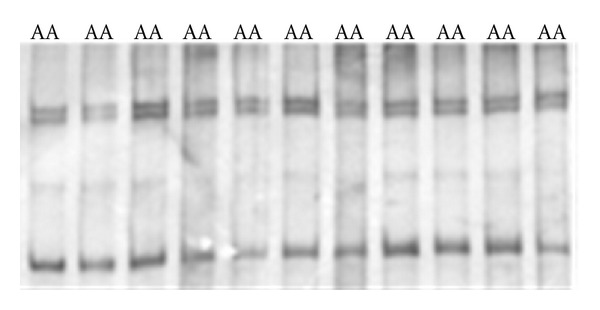
The *SSCP* patterns of intron 1 (222 bp), on 10% nondenatured polyacrylamide gel after silver nitrate staining.

**Figure 2:**
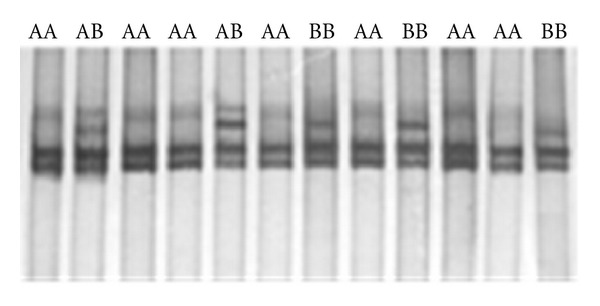
The *SSCP* patterns of intron 2 (311 bp), on 10% nondenatured polyacrylamide gel after silver nitrate staining. Three patterns demonstrating the 3 genotypes are presented.

Genotyping of intron 1 and 2 was performed by *PCR*-*SSCP* method. *PCR* products (3 *μ*L) were diluted with 13 *μ*L of running buffer (including 800 *μ*L formamide 99%, 100 *μ*L loading dye, 100 *μ*L glycerol 98%, 3 *μ*L 0.5M EDTA, and 2 *μ*L 10M NaOH). After heating at 95°C for 5 min, they were immediately placed on ice for 10 min. Polymorphisms were detected using 10% nondenaturing polyacrylamide gels (Figures 1 and 2). The mixture was electrophoresed for 4 h at 250 V and 10°C. DNA fragments were visualized using the silver nitrate staining method [11]. 

### 2.5. Statistical Analysis

Calculation of genotypes, alleles frequencies, mean expected and observed heterozygosities, and Chi-square test was performed using PopGene32 (ver. 1.32) [12]. Samples, which were born in 2006, 2007, and 2008 years, were used for statistical analysis. Yearling weight (*YW*) was analyzed using the fixed model of SAS [13] software and by *GLM* procedure by the following statistical model:

(1) Yijkl=μ+Si+Dj+Gk+eijkl,

where *Y*_*ijkl*_ is yearling weight of each animal; *μ* is general mean; *S*_*i*_ is sex effect (**i* = 1*, and 2), *D*_*j*_ is birth year effect (**j* = 1*, 2, and 3), G_k_ is genotype effect (**k* = 1*, 2, and 3), *e*_*ijkl*_ is random error.

## 3. Results

### 3.1. Exon 3

**Figure 3:**
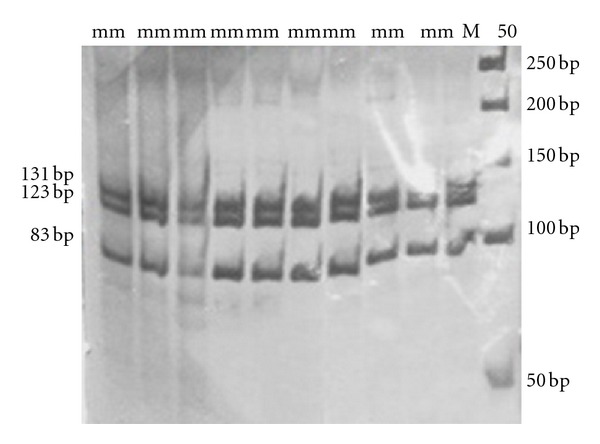
Restriction patterns of 337 bp fragments of exon 3 after digesting with *HaeIII* on 8% nondenatured polyacrylamide gel after silver nitrate staining. Molecular marker was M50.

A 337 bp fragment for exon 3 of *MSTN* locus was amplified. *HaeIII* restriction enzyme was used to digest the *PCR* products. The *HaeIII* digests the *m* allele but not *M* allele. Digestion of the *m* allele produced three fragments of 83, 123, and 131 bp (Figure 3). All samples were digested by *HaeIII* enzyme and showed the *mm* genotype. As a result, all of them were monomorph (Figure 1).

### 3.2. Intron 1 and 2

Intron 1 and 2 of myostatin gene with 222 and 311 bp lengths were amplified, respectively. Under the *SSCP* analysis, different conformations were detected by electrophoresis on 12% nondenaturing polyacrylamide gel (Figure 2). Results showed that intron 1 of this gene was also monomorph, and all samples showed the homozygote genotype (Figure 1). Different conformations were found in intron 2, and *A *and* B* alleles were detected with frequencies of 75.5 and 24.5%, respectively. In this population, this locus did not show Hardy-Weinberg equilibrium (*P* < 0.05) (Table 3). Observed heterozygosity for this locus was very low (0.04) showing high level of homozygosity in the herd. Results showed that there was no significant effect of genotypes of myostatin gene on yearling weights (*P* > 0.05) (Table 4). However, sex had significant effect on *YW *(*P* < 0.01). Yearling weight least squares means of males (31.62 ± 1.34 kg) were more than females (25.21 ± 0.57 kg).

## 4. Discussion

Results showed polymorphism in intron 2, but intron 1 and exon 3 were monomorph. On the contrary, Soufy et al. [14, 15] observed polymorphism for exon 3 in Sanjabi sheep and native Kermanian cattle. Intron 1 was also monomorph, and all samples showed the homozygote genotype. On the other hand, intron 2 was polymorphic and three different genotypes were detected. Three different conformational patterns (*AA, AB, *and* BB*) were determined with frequencies of 73.5, 4, and 22.5%. The allelic frequencies for *A* and *B* were as 75.5 and 24.5%, respectively. Similar result was observed in Iranian Baluchi sheep [16, 17]. This inconsistency may be ascribed to breed differences, population and sampling size, environmental factors, mating strategies, geographical position effect, and frequency distribution of genetic variants.

 Statistical analysis showed that myostatin locus had no significant effect on *YW *(*P* > 0.05). Similar to these findings, Masoudi et al. [17] did not report any significant effect of this locus on *YW*. Although, they found significant effect of different genotypes on birth weight, they also did not observe any significant effect on weaning and six month weights. Ansary et al. [16] detected significant effect of different genotypes on daily gain from birth to 3 month of age (*P* < 0.01). This may be due to the environmental effects that exist and affect this trait. It must be pointed that mutation in intron region is classified as a silent mutation and in spite of existence of mutation in this gene any associations is not reported. However, there are reports of diseases caused by silent mutations. It also seems that introns have a role in the expression of gene and necessitate for physical instructors of DNA. But they do not have a major role in rank of amino acids and proteins' instructor [18].

In this population, this locus did not show Hardy-Weinberg equilibrium. This confirmed that factors leading to disequilibrium, especially selection, may affect the genetic structure of the population. Based on our results, the investigated population showed a low degree of genotypic variability for the *MSTN* gene. This may be explained by the conservation and breeding strategies, which have been carried out. In recent years, in this station, only a few rams have been used as sires in breeding plans. Due to small effective population size, inbreeding was high, and, as a consequence, heterozygosity and genetic variability were low. Controlled breeding might help in lowering inbreeding. In spite of low variability for genomic DNA, these data provide evidence that Iranian's Zel sheep breed have a polymorphism in intron 2 for myostatin locus. However, results showed that this locus in this population may not be useful for developing future selection programs, especially marker-assistant selection for improving weight gain and meat traits.

It can be concluded that, although *MSTN* polymorphism did not have effect on *YW*, further analysis needs to be conducted on the effect of *MSTN* genotypes on yearling weight and other body weights. Furthermore, results showed that *PCR*-*RFLP* and *PCR*-*SSCP* are appropriate tools for evaluating genetic variability.

## Figures and Tables

**Table 1:** Region, methods, primer's sequence (5^'^→3^'^), and length of *PCR* products of the ovine myostatin gene.

Region	Using method	Primer's sequence (5^'^→3^'^)	Length of fragment (bp)
Intron 1	*PCR-SSCP*	F: TAC CTT CAT CAC TCT GCC TTC C	222
		R: GGA GGA AAG AAG AGG GAC AAG	
Intron 2	*PCR-SSCP*	F: CAC ATT TTT CCC CCA GAA GAG	311
		R: AAG ACA GTT CAG AAA ATA GCT GG	
Exon 3	*PCR-RFLP*	F: CCG GAG AGA CTT TGG GCT TGA	337
		R: TCA TGA GCA CCC ACA GCG GTC	

F: forward and R: reverse.

**Table 2:** *PCR* conditions.

Location	Primary denaturation in 1st cycle	Denaturation		Annealing		Elongation		Final extension	Number of cycles
	^ °^C/Sec	^ °^C	Sec	^ °^C	Sec	^ °^C	Sec	^ °^C/Sec	*n*
Intron 1	95/240	94	60	56.5	70	72	75	72/600	40
Intron 2	95/240	95	50	55	60	72	75	72/600	40
Exon 3	94/240	94	60	58.5	60	72	120	72/240	35

**Table 3:** Allele and genotype frequencies, observed, expected, and average heterozygosity for intron 2 of *MSTN* gene.

Locus	Allelic frequencies (%)		Genotype frequencies (%)			Heterozygosity			*χ* ^2^
	*A*	*B*	*AA*	*AB*	*BB*	Obs.	Exp.	Ave.
Intron 2	75.5	24.5	73.5	4	22.5	0.04	0.37	0.37	160.55^∗^

*P < 0.05.

**Table 4:** Least square means (*LSM*), standard error (*SE*), and probability levels for *YW* (kg) of intron 2 of *MSTN* genotypes.

Probability levels			Genotype	LSM^∗^ ± SE
*AA*	*AB*	*BB*
—	0.9539	0.6367	*AA*	28.30^a^ ± 0.72
0.9539	—	0.7397	*AB*	27.85^a^ ± 1.61
0.6367	0.7397	—	*BB*	29.09^a^ ± 0.84

^∗^Same letters in column show no significant difference (*P* > 0.05).
